# 

**Published:** 1876-06

**Authors:** 


*CONTENTS.*

| | Page |
|---|---|
| Portrait—Dr. W. W. Hall | 241 |
| Obituary | 243 |
| Death's Summer Campaign | 245 |
| In Poor Health | 246 |
| Bad Cooking | 247 |
| Health and Capital | 248 |
| Homes for Invalids | 249 |
| An Explanation | 250 |
| Announcement | 250 |
| The Atheist (Poetry) | 251 |
| Communication | 252 |
| Egyptian Fellahs | 252 |
| A Good Fire Insurance Co. | 253 |
| Treatment of Cancer | 254 |
| New Publications | 255 |
| Antidote for Poisons | 256 |
| The Poetry of Poverty | 257 |
| Good-Night (Poetry) | 260 |
| War | 260 |
| Out of Evil, Good | 265 |
| Themes for the Thoughtful | 270 |
| Eating and Exercise | 273 |
| Donation Parties | 274 |
| Whisky Doctors | 274 |
| Position in Sleeping | 275 |
| Sociability | 276 |
| A Chat about Health | 277 |
| Taking Medicine | 278 |
| Destructive Agencies | 279 |
| School Outrages | 279 |
| A New Duty | 280 |
| Gloved to Death | 281 |
| Cholera Infantum | 281 |
| Food | 282 |
| Hydropathy | 283 |
| In the Long Run | 283 |

